# Crystal structure of 3-amino-1-(4-chloro­phen­yl)-1*H*-benzo[*f*]chromene-2-carbo­nitrile

**DOI:** 10.1107/S2056989015011159

**Published:** 2015-06-13

**Authors:** Mehmet Akkurt, Peter N. Horton, Shaaban K. Mohamed, Sabry H. H. Younes, Mustafa R. Albayati

**Affiliations:** a Department of Physics, Faculty of Sciences, Erciyes University, 38039 Kayseri, Turkey; b School of Chemistry, University of Southampton, Highfield, Southampton SO17 1BJ, England; c Chemistry and Environmental Division, Manchester Metropolitan University, Manchester M1 5GD, England; d Chemistry Department, Faculty of Science, Minia University, 61519 El-Minia, Egypt; e Chemistry Department, Faculty of Science, Sohag University, 82524 Sohag, Egypt; f Kirkuk University, College of Science, Department of Chemistry, Kirkuk, Iraq

**Keywords:** crystal structure, chromene compounds, N—H⋯N hydrogen bonds, C—H⋯π inter­actions

## Abstract

In the title compound, C_20_H_13_ClN_2_O, the chloro­benzene ring is almost perpendicular to the mean plane of the naphthalene ring system, making a dihedral angle of 81.26 (8)°. The 4*H*-pyran ring fused with the naphthalene ring system has a flattened boat conformation. In the crystal, N—H⋯N hydrogen bonds generate chains along the *b-*axis direction. Further N—H⋯N hydrogen bonds link these chains into sheets parallel to (010). The crystal packing also features C—H⋯π inter­actions. The crystal studied was an inversion twin with a 0.557 (16):0.443 (16) domain ratio.

## Related literature   

For the synthesis and biological importance of chromene compounds, see, for example: Ellis (1977 ▸); Singh *et al.* (2010 ▸); Kidwai *et al.* (2010 ▸); Lácová *et al.* (2005 ▸); Dell & Smith (1993*a* ▸,*b* ▸); Al-Soud *et al.* (2006 ▸); Eiden & Denk (1991 ▸); Bruhlmann *et al.* (200); (Kesten *et al.* (1999 ▸); Bruhlmann *et al.* (2001 ▸). For a similar structure, see: Akkurt *et al.* (2013 ▸).
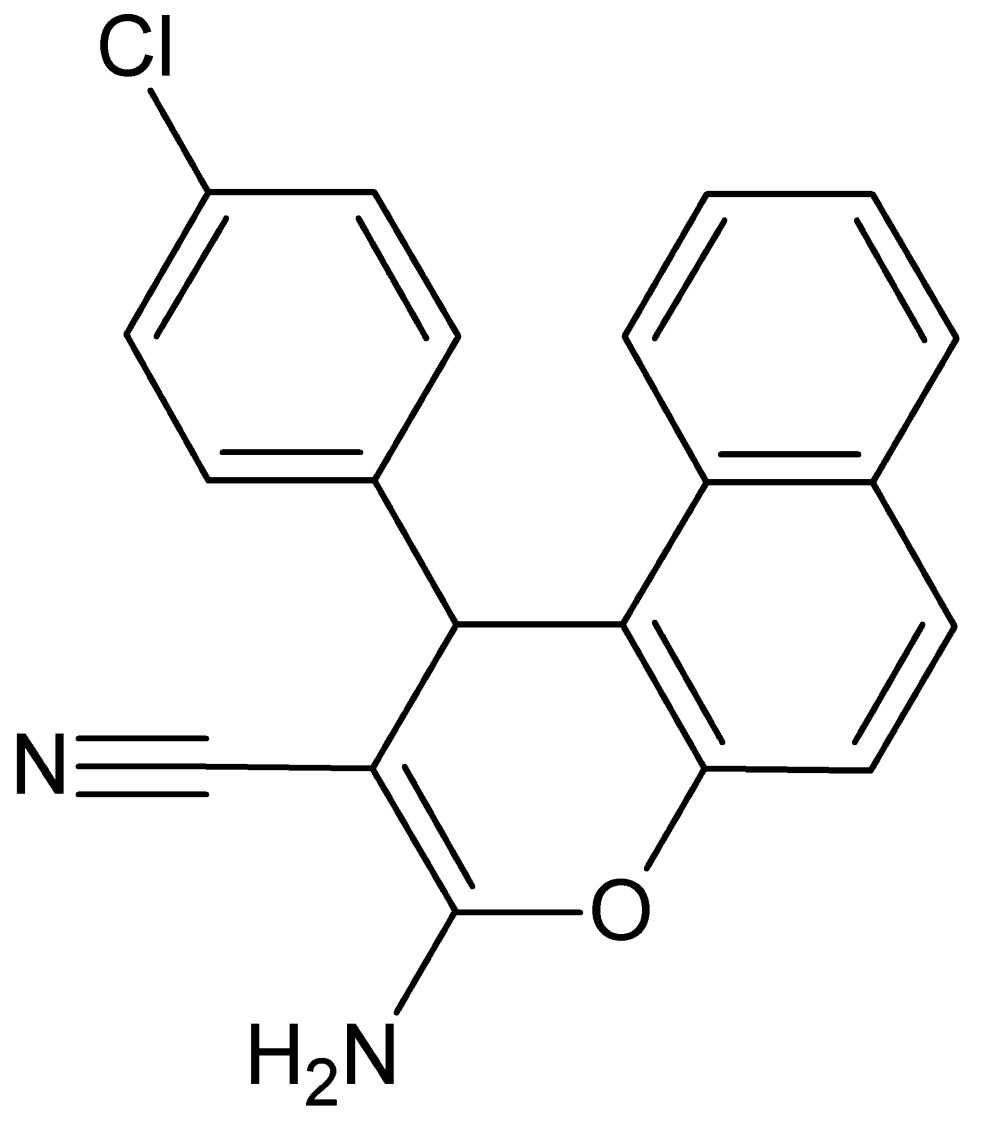


## Experimental   

### Crystal data  

C_20_H_13_ClN_2_O
*M* *_r_* = 332.77
Monoclinic, 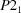

*a* = 10.056 (7) Å
*b* = 6.172 (3) Å
*c* = 12.751 (6) Å
β = 99.641 (17)°
*V* = 780.2 (8) Å^3^
*Z* = 2
Cu *K*α radiation
μ = 2.23 mm^−1^
*T* = 100 K
0.32 × 0.12 × 0.10 mm

### Data collection  

Rigaku AFC11 diffractometer
Absorption correction: multi-scan (*CrystalClearSM Expert*; Rigaku, 2012 ▸) *T*_min_ = 0.948, *T*_max_ = 1.000
11415 measured reflections
2558 independent reflections
2500 reflections with *I* > 2σ(*I*)
*R*_int_ = 0.032

### Refinement  

*R*[*F* ^2^ > 2σ(*F* ^2^)] = 0.028
*wR*(*F* ^2^) = 0.075
*S* = 1.06
2558 reflections
219 parameters
1 restraint
H-atom parameters constrained
Δρ_max_ = 0.17 e Å^−3^
Δρ_min_ = −0.22 e Å^−3^
Absolute structure: Refined as an inversion twin.
Absolute structure parameter: 0.443 (16)

### 

Data collection: *CrystalClearSM Expert* (Rigaku, 2012 ▸); cell refinement: *CrystalClearSM Expert*; data reduction: *CrystalClearSM Expert*; program(s) used to solve structure: *SUPERFLIP* (Palatinus & Chapuis, 2007 ▸); program(s) used to refine structure: *SHELXL2014* (Sheldrick, 2015 ▸); molecular graphics: *ORTEP-3 for Windows* (Farrugia, 2012 ▸); software used to prepare material for publication: *WinGX* (Farrugia, 2012 ▸).

## Supplementary Material

Crystal structure: contains datablock(s) global, I. DOI: 10.1107/S2056989015011159/sj5465sup1.cif

Structure factors: contains datablock(s) I. DOI: 10.1107/S2056989015011159/sj5465Isup2.hkl

Supporting information file. DOI: 10.1107/S2056989015011159/sj5465Isup3.cml

**Fig. 1.**
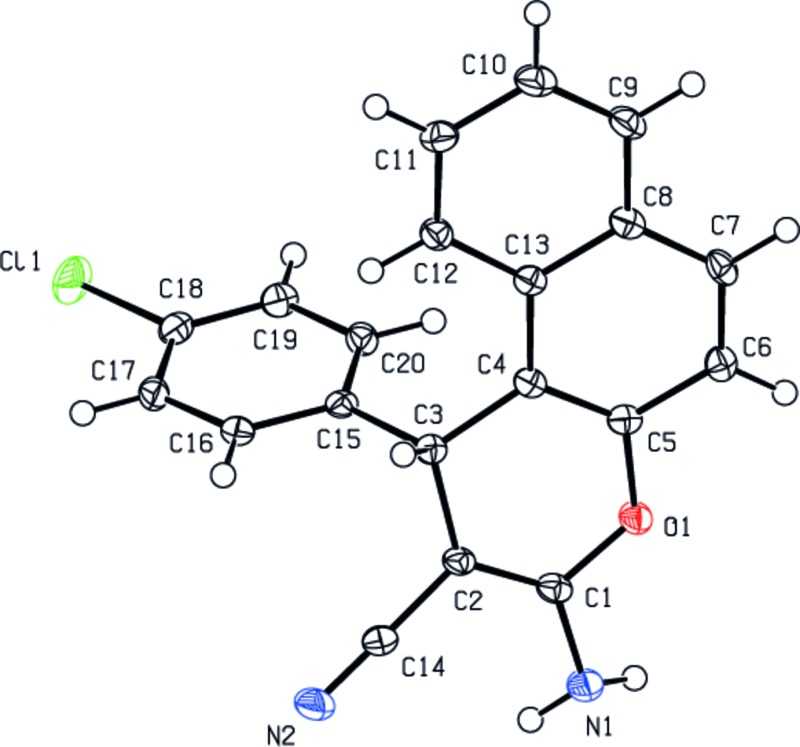
View of the title compound with the atom-numbering scheme. Displacement ellipsoids for non-H atoms are drawn at the 50% probability level.

**Fig. 2.**
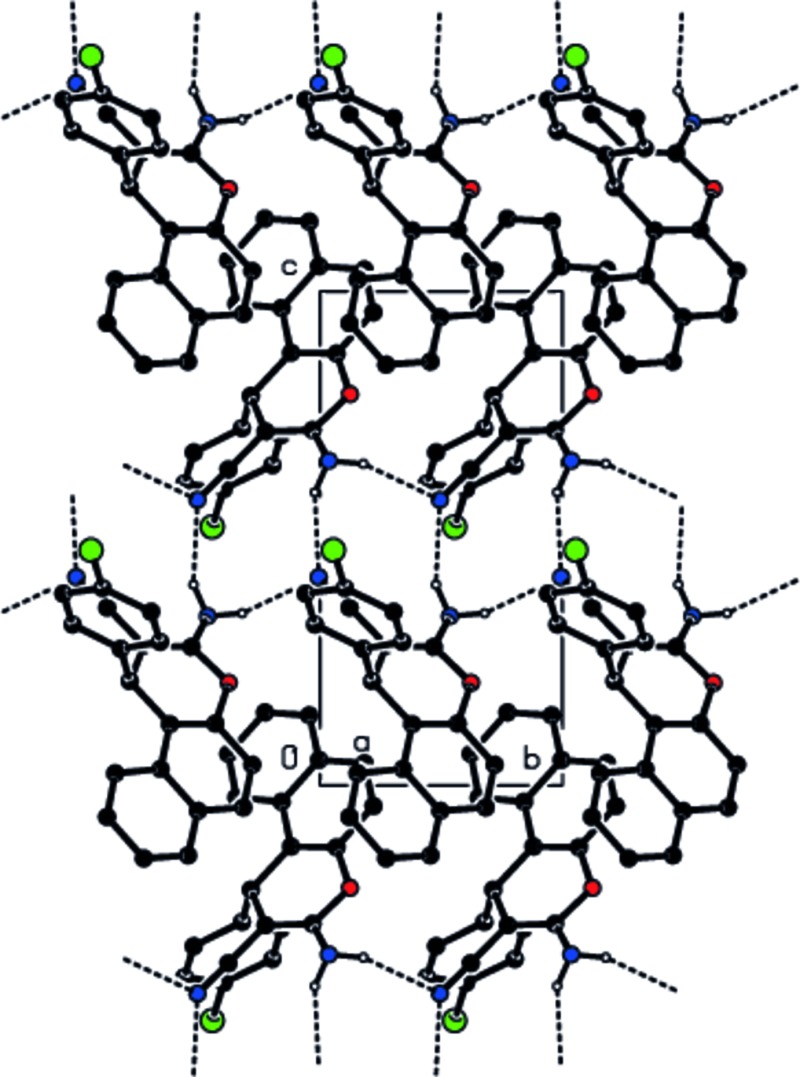
Crystal packing of the title compound viewed along the a axis, with hydrogen bonds drawn as dashed lines.

**Fig. 3.**
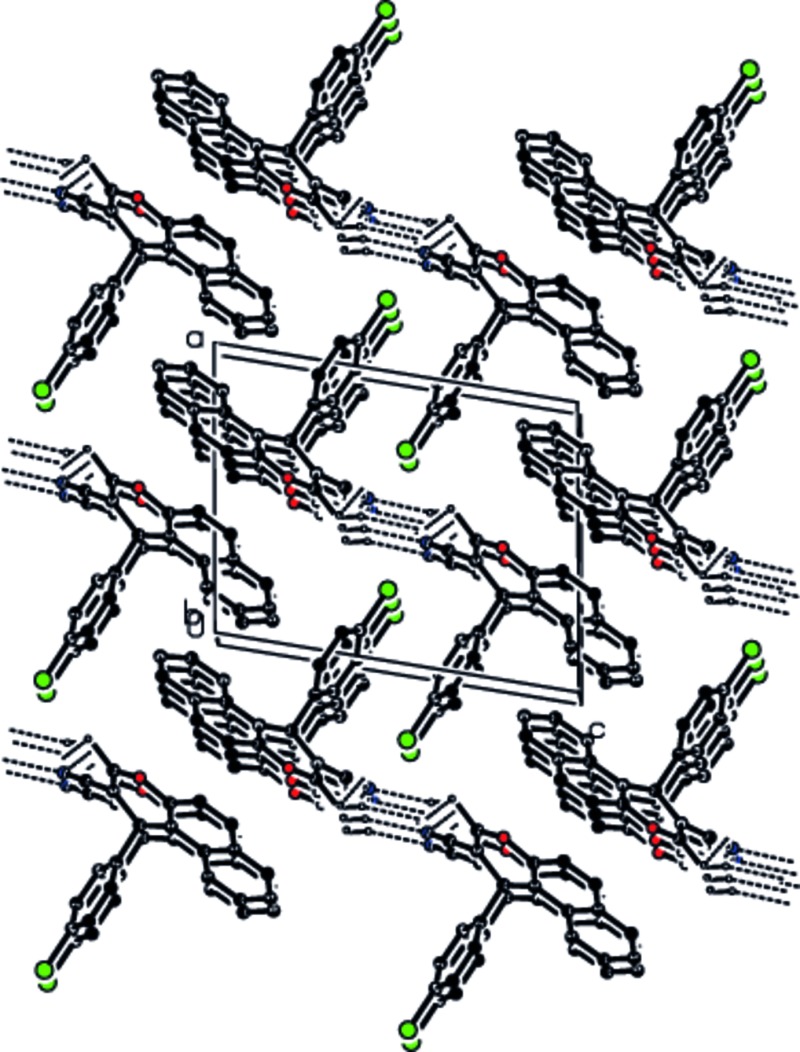
A view of the packing showing molecules stacked along the b axis.

. DOI: 10.1107/S2056989015011159/sj5465fig1.tif
View of the title compound with the atom-numbering scheme. Displacement ellipsoids for non-H atoms are drawn at the 50% probability level.

a . DOI: 10.1107/S2056989015011159/sj5465fig2.tif
Crystal packing of the title compound viewed along the *a* axis, with hydrogen bonds drawn as dashed lines.

b . DOI: 10.1107/S2056989015011159/sj5465fig3.tif
A view of the packing showing mol­ecules stacked along the *b* axis.

CCDC reference: 1405640

Additional supporting information:  crystallographic information; 3D view; checkCIF report

## Figures and Tables

**Table 1:** Hydrogen-bond geometry (, ) *Cg*2 and *Cg*3 are the centroids of the C4C8/C13 and C8C13 rings, respectively.

*D*H*A*	*D*H	H*A*	*D* *A*	*D*H*A*
N1H1*A*N2^i^	0.88	2.22	3.005(3)	148
N1H1*B*N2^ii^	0.88	2.32	3.129(4)	152
C6H6*Cg*2^iii^	0.95	2.60	3.401(3)	142
C11H11*Cg*3^iv^	0.95	2.90	3.636(3)	135

Symmetry codes: (i) 
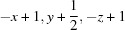
; (ii) 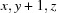; (iii) 
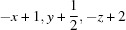
; (iv) 
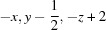
.

## supplementary crystallographic information

### S1. Comment

The chromene moiety is found in natural products and exhibits various biological activities (Ellis, 1977; Singh *et al.*, 2010; Lácová *et al.*, 2010; Kidwai *et al.*, 2005). Fused chromene derivatives are biologically interesting compounds showing antiproliferation activity (Dell & Smith, 1993*a*,*b*), are effective anti-HIV agents (Al-Soud *et al.*, 2006) and impact the central nervous system (*CNS*) (Eiden & Denk, 1991). Moreover, they have been employed in the treatment of Alzheimer's disease (Bruhlmann *et al.*, 2001) and the Schizophrenia disorder (Kesten *et al.*, 1999). In this context, we report in this study the synthesis and crystal structure of the title compound.

In the title compound (Fig. 1), the choloro-benzene ring (C15–C20) is approximately perpendicular to the naphthalene ring system [C4–C13, maximum deviation = 0.012 (2) Å at atom C4] as indicated by the dihedral angle of 81.26 (5)°. The 4*H*-pyran ring (O1/C1–C5) in the title compound is puckered with the puckering parameters of Q_T_ = 0.143 (2) Å, θ = 86.4 (8)° and φ = 167.3 (9)°. All the bond lengths and angles in the title compound are within normal ranges and comparable with those reported for a similar structure (Akkurt *et al.*, 2013).

In the crystal structure, molecules are linked into sheets parallel to (010) by the N1—H1A···N2 and N1—H1B···N2 hydrogen bonds (Table 1, Figs 2 and 3), which generate chains along [010]. The crystal packing is further stabilized by C—H···π interactions (Table 1).

### S2. Experimental

A mixture of 4-chlorobenzylidenepropanedinitrile (188.5 mg; 1 mmol) and 2-naphthol (144 mg; 1 mmol) was refluxed with stirring for 2 h at 350 K in ethanol (10 ml) in the presence of a catalytic amount of triethylamine. After cooling to room temperature, the solid product was collected by filtration, washed with cold ethanol and dried under vacuum. High quality crystals suitable for X-ray diffraction were obtained in an excellent yield (96%) by recrystallization of the crude product from ethanol. M.p. K.

### S3. Refinement

All H atoms were placed geometrically and treated as riding on their parent atoms with N—H = 0.88 Å, C—H = 0.95 Å (aromatic CH), C—H = 1.00 Å (methine CH), and with *U*_iso_(H) = 1.2*U*_eq_(C,*N*). The crystal studied was an inversion twin with a 0.557 (16):0.443 (16) domain ratio.

### Crystal data

**Table:**

C_20_H_13_ClN_2_O	*F*(000) = 344
*M**_r_* = 332.77	*D*_x_ = 1.416 Mg m^−^^3^
Monoclinic, *P*2_1_	Cu *K*α radiation, λ = 1.54178 Å
Hall symbol: P 2yb	Cell parameters from 1130 reflections
*a* = 10.056 (7) Å	θ = 21.5–68.7°
*b* = 6.172 (3) Å	µ = 2.23 mm^−^^1^
*c* = 12.751 (6) Å	*T* = 100 K
β = 99.641 (17)°	Block, light brown
*V* = 780.2 (8) Å^3^	0.32 × 0.12 × 0.10 mm
*Z* = 2	

### Data collection

**Table:**

Rigaku AFC11 diffractometer	2558 independent reflections
Radiation source: Rotating Anode	2500 reflections with *I* > 2σ(*I*)
Detector resolution: 22.2222 pixels mm^-1^	*R*_int_ = 0.032
profile data from ω–scans	θ_max_ = 66.6°, θ_min_ = 3.5°
Absorption correction: multi-scan (*CrystalClearSM Expert*; Rigaku, 2012)	*h* = −11→11
*T*_min_ = 0.948, *T*_max_ = 1.000	*k* = −7→7
11415 measured reflections	*l* = −15→15

### Refinement

**Table:**

Refinement on *F*^2^	Hydrogen site location: mixed
Least-squares matrix: full	H-atom parameters constrained
*R*[*F*^2^ > 2σ(*F*^2^)] = 0.028	*w* = 1/[σ^2^(*F*_o_^2^) + (0.0548*P*)^2^ + 0.1146*P*] where *P* = (*F*_o_^2^ + 2*F*_c_^2^)/3
*wR*(*F*^2^) = 0.075	(Δ/σ)_max_ < 0.001
*S* = 1.06	Δρ_max_ = 0.17 e Å^−^^3^
2558 reflections	Δρ_min_ = −0.22 e Å^−^^3^
219 parameters	Absolute structure: Refined as an inversion twin.
1 restraint	Absolute structure parameter: 0.443 (16)

### Special details

**Table:**

Geometry. Bond distances, angles *etc*. have been calculated using the rounded fractional coordinates. All su's are estimated from the variances of the (full) variance-covariance matrix. The cell e.s.d.'s are taken into account in the estimation of distances, angles and torsion angles
Refinement. Refinement on *F*^2^ for ALL reflections except those flagged by the user for potential systematic errors. Weighted *R*-factors *wR* and all goodnesses of fit *S* are based on *F*^2^, conventional *R*-factors *R* are based on *F*, with *F* set to zero for negative *F*^2^. The observed criterion of *F*^2^ > σ(*F*^2^) is used only for calculating -*R*-factor-obs *etc*. and is not relevant to the choice of reflections for refinement. *R*-factors based on *F*^2^ are statistically about twice as large as those based on *F*, and *R*-factors based on ALL data will be even larger.

### Fractional atomic coordinates and isotropic or equivalent isotropic displacement parameters (Å^2^)

**Table:**

	*x*	*y*	*z*	*U*_iso_*/*U*_eq_	
Cl1	−0.24323 (5)	0.55572 (12)	0.52362 (5)	0.0334 (2)	
O1	0.45126 (15)	1.1239 (3)	0.79232 (11)	0.0189 (5)	
N1	0.55640 (19)	1.0368 (4)	0.65748 (14)	0.0214 (6)	
N2	0.4506 (2)	0.4950 (3)	0.57819 (16)	0.0257 (6)	
C1	0.4675 (2)	0.9698 (4)	0.71956 (16)	0.0166 (6)	
C2	0.4046 (2)	0.7754 (4)	0.71666 (16)	0.0156 (6)	
C3	0.3043 (2)	0.7152 (4)	0.78878 (16)	0.0149 (6)	
C4	0.3101 (2)	0.8809 (4)	0.87613 (16)	0.0144 (6)	
C5	0.38290 (19)	1.0685 (4)	0.87480 (15)	0.0156 (6)	
C6	0.3992 (2)	1.2209 (4)	0.95817 (16)	0.0174 (6)	
C7	0.3399 (2)	1.1839 (4)	1.04481 (17)	0.0180 (6)	
C8	0.2613 (2)	0.9965 (4)	1.05160 (17)	0.0175 (6)	
C9	0.1991 (2)	0.9560 (4)	1.14178 (17)	0.0212 (7)	
C10	0.1240 (2)	0.7733 (5)	1.14784 (18)	0.0240 (7)	
C11	0.1072 (2)	0.6218 (4)	1.06388 (17)	0.0214 (7)	
C12	0.1664 (2)	0.6555 (4)	0.97558 (17)	0.0175 (6)	
C13	0.24556 (19)	0.8423 (4)	0.96678 (16)	0.0149 (6)	
C14	0.4305 (2)	0.6224 (4)	0.63990 (16)	0.0179 (6)	
C15	0.1641 (2)	0.6784 (4)	0.72333 (16)	0.0150 (6)	
C16	0.1348 (2)	0.4796 (4)	0.67440 (16)	0.0174 (6)	
C17	0.0094 (2)	0.4409 (4)	0.61165 (17)	0.0202 (7)	
C18	−0.0858 (2)	0.6031 (4)	0.60027 (16)	0.0204 (7)	
C19	−0.0595 (2)	0.8037 (4)	0.64830 (17)	0.0201 (7)	
C20	0.0667 (2)	0.8403 (4)	0.70949 (16)	0.0176 (6)	
H1A	0.53650	0.97850	0.59340	0.0260*	
H1B	0.55280	1.17950	0.65130	0.0260*	
H3	0.33450	0.57400	0.82330	0.0180*	
H6	0.45110	1.34820	0.95390	0.0210*	
H7	0.35150	1.28550	1.10150	0.0220*	
H9	0.20980	1.05740	1.19860	0.0250*	
H10	0.08300	0.74830	1.20870	0.0290*	
H11	0.05460	0.49540	1.06840	0.0260*	
H12	0.15400	0.55200	0.91960	0.0210*	
H16	0.20090	0.36820	0.68370	0.0210*	
H17	−0.00980	0.30510	0.57740	0.0240*	
H19	−0.12630	0.91410	0.63960	0.0240*	
H20	0.08640	0.97750	0.74210	0.0210*	

### Atomic displacement parameters (Å^2^)

**Table:**

	*U*^11^	*U*^22^	*U*^33^	*U*^12^	*U*^13^	*U*^23^
Cl1	0.0198 (3)	0.0457 (4)	0.0312 (3)	−0.0081 (3)	−0.0061 (2)	−0.0053 (3)
O1	0.0234 (8)	0.0193 (9)	0.0146 (7)	−0.0026 (6)	0.0054 (6)	−0.0006 (6)
N1	0.0250 (9)	0.0222 (11)	0.0189 (9)	−0.0033 (9)	0.0088 (7)	0.0002 (8)
N2	0.0313 (11)	0.0258 (12)	0.0233 (10)	−0.0026 (9)	0.0146 (8)	−0.0028 (9)
C1	0.0160 (10)	0.0226 (12)	0.0110 (9)	0.0030 (9)	0.0016 (8)	0.0005 (8)
C2	0.0135 (10)	0.0219 (12)	0.0113 (9)	0.0010 (9)	0.0019 (7)	−0.0013 (8)
C3	0.0146 (11)	0.0167 (12)	0.0132 (9)	0.0009 (8)	0.0020 (8)	0.0009 (8)
C4	0.0122 (9)	0.0183 (11)	0.0117 (10)	0.0041 (9)	−0.0009 (7)	0.0001 (8)
C5	0.0139 (9)	0.0206 (12)	0.0118 (9)	0.0024 (9)	0.0010 (7)	0.0023 (9)
C6	0.0152 (11)	0.0182 (12)	0.0172 (10)	0.0019 (8)	−0.0021 (8)	−0.0006 (9)
C7	0.0190 (11)	0.0195 (12)	0.0137 (9)	0.0032 (9)	−0.0021 (8)	−0.0044 (9)
C8	0.0158 (10)	0.0228 (13)	0.0131 (9)	0.0045 (9)	0.0000 (8)	−0.0005 (8)
C9	0.0228 (12)	0.0261 (13)	0.0152 (10)	0.0025 (10)	0.0044 (9)	−0.0047 (10)
C10	0.0257 (13)	0.0302 (14)	0.0181 (11)	0.0012 (11)	0.0091 (9)	−0.0003 (10)
C11	0.0201 (11)	0.0249 (13)	0.0207 (11)	−0.0013 (9)	0.0078 (9)	0.0004 (9)
C12	0.0169 (10)	0.0192 (12)	0.0163 (10)	0.0012 (9)	0.0021 (8)	−0.0017 (9)
C13	0.0125 (10)	0.0176 (12)	0.0138 (10)	0.0035 (8)	0.0003 (8)	0.0006 (8)
C14	0.0176 (10)	0.0200 (13)	0.0170 (10)	−0.0005 (8)	0.0058 (8)	0.0019 (9)
C15	0.0151 (10)	0.0213 (12)	0.0089 (9)	−0.0017 (9)	0.0032 (7)	0.0011 (8)
C16	0.0180 (11)	0.0197 (12)	0.0155 (10)	−0.0005 (9)	0.0056 (8)	−0.0008 (9)
C17	0.0242 (12)	0.0228 (13)	0.0145 (10)	−0.0083 (10)	0.0060 (8)	−0.0034 (9)
C18	0.0165 (10)	0.0325 (15)	0.0119 (9)	−0.0063 (9)	0.0018 (8)	−0.0008 (9)
C19	0.0179 (11)	0.0248 (13)	0.0175 (11)	0.0003 (10)	0.0023 (8)	0.0016 (9)
C20	0.0189 (11)	0.0187 (12)	0.0149 (10)	−0.0007 (9)	0.0022 (8)	−0.0011 (9)

### Geometric parameters (Å, º)

**Table:**

Cl1—C18	1.740 (2)	C10—C11	1.410 (4)
O1—C1	1.358 (3)	C11—C12	1.375 (3)
O1—C5	1.392 (3)	C12—C13	1.416 (3)
N1—C1	1.354 (3)	C15—C20	1.390 (3)
N2—C14	1.154 (3)	C15—C16	1.386 (3)
C1—C2	1.354 (3)	C16—C17	1.396 (3)
N1—H1B	0.8800	C17—C18	1.376 (3)
N1—H1A	0.8800	C18—C19	1.387 (4)
C2—C14	1.415 (3)	C19—C20	1.392 (3)
C2—C3	1.520 (3)	C3—H3	1.0000
C3—C4	1.506 (3)	C6—H6	0.9500
C3—C15	1.530 (3)	C7—H7	0.9500
C4—C13	1.437 (3)	C9—H9	0.9500
C4—C5	1.372 (3)	C10—H10	0.9500
C5—C6	1.408 (3)	C11—H11	0.9500
C6—C7	1.360 (3)	C12—H12	0.9500
C7—C8	1.412 (3)	C16—H16	0.9500
C8—C13	1.429 (3)	C17—H17	0.9500
C8—C9	1.420 (3)	C19—H19	0.9500
C9—C10	1.367 (4)	C20—H20	0.9500
			
C1—O1—C5	118.39 (19)	C3—C15—C20	121.9 (2)
O1—C1—N1	110.6 (2)	C16—C15—C20	119.17 (19)
O1—C1—C2	122.03 (19)	C15—C16—C17	120.9 (2)
N1—C1—C2	127.3 (2)	C16—C17—C18	118.8 (2)
H1A—N1—H1B	109.00	Cl1—C18—C19	119.09 (17)
C1—N1—H1A	110.00	Cl1—C18—C17	119.23 (18)
C1—N1—H1B	110.00	C17—C18—C19	121.7 (2)
C1—C2—C3	123.7 (2)	C18—C19—C20	118.7 (2)
C1—C2—C14	118.10 (19)	C15—C20—C19	120.8 (2)
C3—C2—C14	118.2 (2)	C2—C3—H3	107.00
C2—C3—C4	109.51 (19)	C4—C3—H3	107.00
C4—C3—C15	114.97 (18)	C15—C3—H3	107.00
C2—C3—C15	110.51 (17)	C5—C6—H6	120.00
C5—C4—C13	117.6 (2)	C7—C6—H6	120.00
C3—C4—C5	121.25 (18)	C6—C7—H7	120.00
C3—C4—C13	121.0 (2)	C8—C7—H7	120.00
O1—C5—C4	123.29 (19)	C8—C9—H9	120.00
O1—C5—C6	113.4 (2)	C10—C9—H9	120.00
C4—C5—C6	123.33 (19)	C9—C10—H10	120.00
C5—C6—C7	119.3 (2)	C11—C10—H10	120.00
C6—C7—C8	120.9 (2)	C10—C11—H11	120.00
C9—C8—C13	119.2 (2)	C12—C11—H11	120.00
C7—C8—C9	121.4 (2)	C11—C12—H12	120.00
C7—C8—C13	119.43 (19)	C13—C12—H12	120.00
C8—C9—C10	120.9 (2)	C15—C16—H16	120.00
C9—C10—C11	120.1 (2)	C17—C16—H16	120.00
C10—C11—C12	120.6 (2)	C16—C17—H17	121.00
C11—C12—C13	121.0 (2)	C18—C17—H17	121.00
C8—C13—C12	118.33 (19)	C18—C19—H19	121.00
C4—C13—C12	122.3 (2)	C20—C19—H19	121.00
C4—C13—C8	119.4 (2)	C15—C20—H20	120.00
N2—C14—C2	178.9 (2)	C19—C20—H20	120.00
C3—C15—C16	119.0 (2)		
			
C5—O1—C1—N1	−168.57 (17)	C13—C4—C5—O1	179.31 (19)
C5—O1—C1—C2	8.9 (3)	O1—C5—C6—C7	−178.62 (19)
C1—O1—C5—C6	165.88 (18)	C4—C5—C6—C7	−0.4 (3)
C1—O1—C5—C4	−12.3 (3)	C5—C6—C7—C8	−0.7 (3)
O1—C1—C2—C14	−178.26 (19)	C6—C7—C8—C9	179.9 (2)
O1—C1—C2—C3	3.8 (3)	C6—C7—C8—C13	0.9 (3)
N1—C1—C2—C14	−1.2 (3)	C7—C8—C9—C10	−179.5 (2)
N1—C1—C2—C3	−179.1 (2)	C13—C8—C9—C10	−0.5 (3)
C1—C2—C3—C15	115.5 (2)	C7—C8—C13—C4	0.0 (3)
C1—C2—C3—C4	−12.1 (3)	C7—C8—C13—C12	179.9 (2)
C14—C2—C3—C4	169.96 (19)	C9—C8—C13—C4	−179.0 (2)
C14—C2—C3—C15	−62.4 (3)	C9—C8—C13—C12	0.9 (3)
C2—C3—C4—C5	8.7 (3)	C8—C9—C10—C11	−0.1 (3)
C15—C3—C4—C13	67.1 (3)	C9—C10—C11—C12	0.3 (3)
C2—C3—C15—C16	82.1 (3)	C10—C11—C12—C13	0.1 (3)
C2—C3—C15—C20	−96.3 (2)	C11—C12—C13—C4	179.2 (2)
C4—C3—C15—C16	−153.4 (2)	C11—C12—C13—C8	−0.7 (3)
C4—C3—C15—C20	28.3 (3)	C3—C15—C16—C17	−178.31 (19)
C2—C3—C4—C13	−167.82 (19)	C20—C15—C16—C17	0.1 (3)
C15—C3—C4—C5	−116.4 (2)	C3—C15—C20—C19	179.08 (19)
C3—C4—C5—O1	2.7 (3)	C16—C15—C20—C19	0.7 (3)
C13—C4—C5—C6	1.3 (3)	C15—C16—C17—C18	−0.9 (3)
C3—C4—C13—C8	175.60 (19)	C16—C17—C18—Cl1	−179.20 (16)
C3—C4—C13—C12	−4.3 (3)	C16—C17—C18—C19	0.8 (3)
C5—C4—C13—C8	−1.1 (3)	Cl1—C18—C19—C20	−180.00 (17)
C5—C4—C13—C12	179.1 (2)	C17—C18—C19—C20	−0.1 (3)
C3—C4—C5—C6	−175.4 (2)	C18—C19—C20—C15	−0.7 (3)

### Hydrogen-bond geometry (Å, º)

Cg2 and Cg3 are the centroids of the C4–C8/C13 and C8–C13 rings, respectively.

**Table:**

*D*—H···*A*	*D*—H	H···*A*	*D*···*A*	*D*—H···*A*
N1—H1*A*···N2^i^	0.88	2.22	3.005 (3)	148
N1—H1*B*···N2^ii^	0.88	2.32	3.129 (4)	152
C6—H6···*Cg*2^iii^	0.95	2.60	3.401 (3)	142
C11—H11···*Cg*3^iv^	0.95	2.90	3.636 (3)	135

Symmetry codes: (i) −*x*+1, *y*+1/2, −*z*+1; (ii) *x*, *y*+1, *z*; (iii) −*x*+1, *y*+1/2, −*z*+2; (iv) −*x*, *y*−1/2, −*z*+2.
