# Supplementary figures and images for: Crystal structure of 3-amino-1-(4-chloro­phen­yl)-1H-benzo[f]chromene-2-carbo­nitrile
Source: Acta Crystallogr E Crystallogr Commun. 2015 Jun 13;71(Pt 7):o481–2. doi: 10.1107/S2056989015011159 (PMC4518915; doi:10.1107/S2056989015011159)

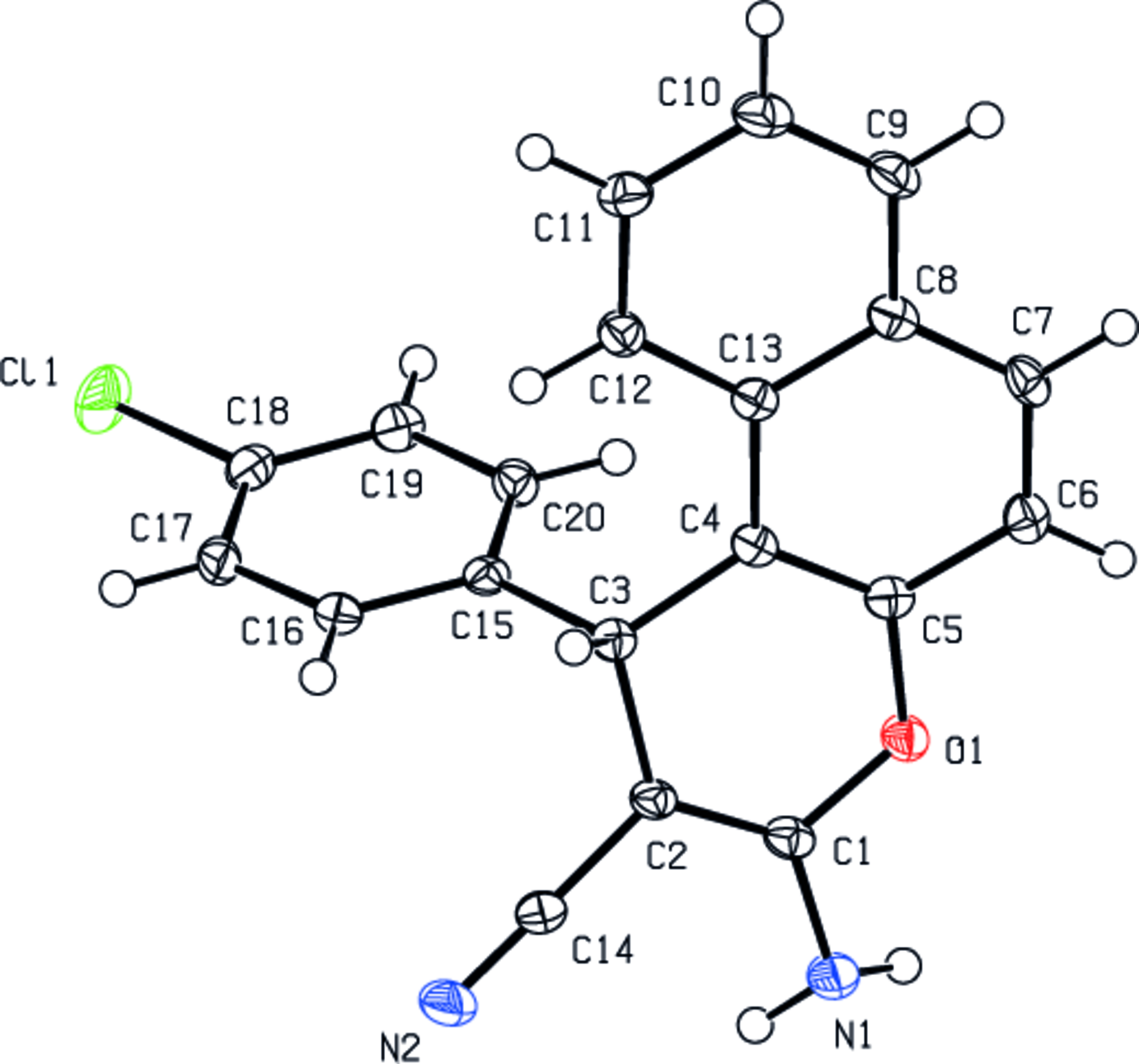

Supplement: Supplementary file 4 [file e-71-0o481-fig1.tif]

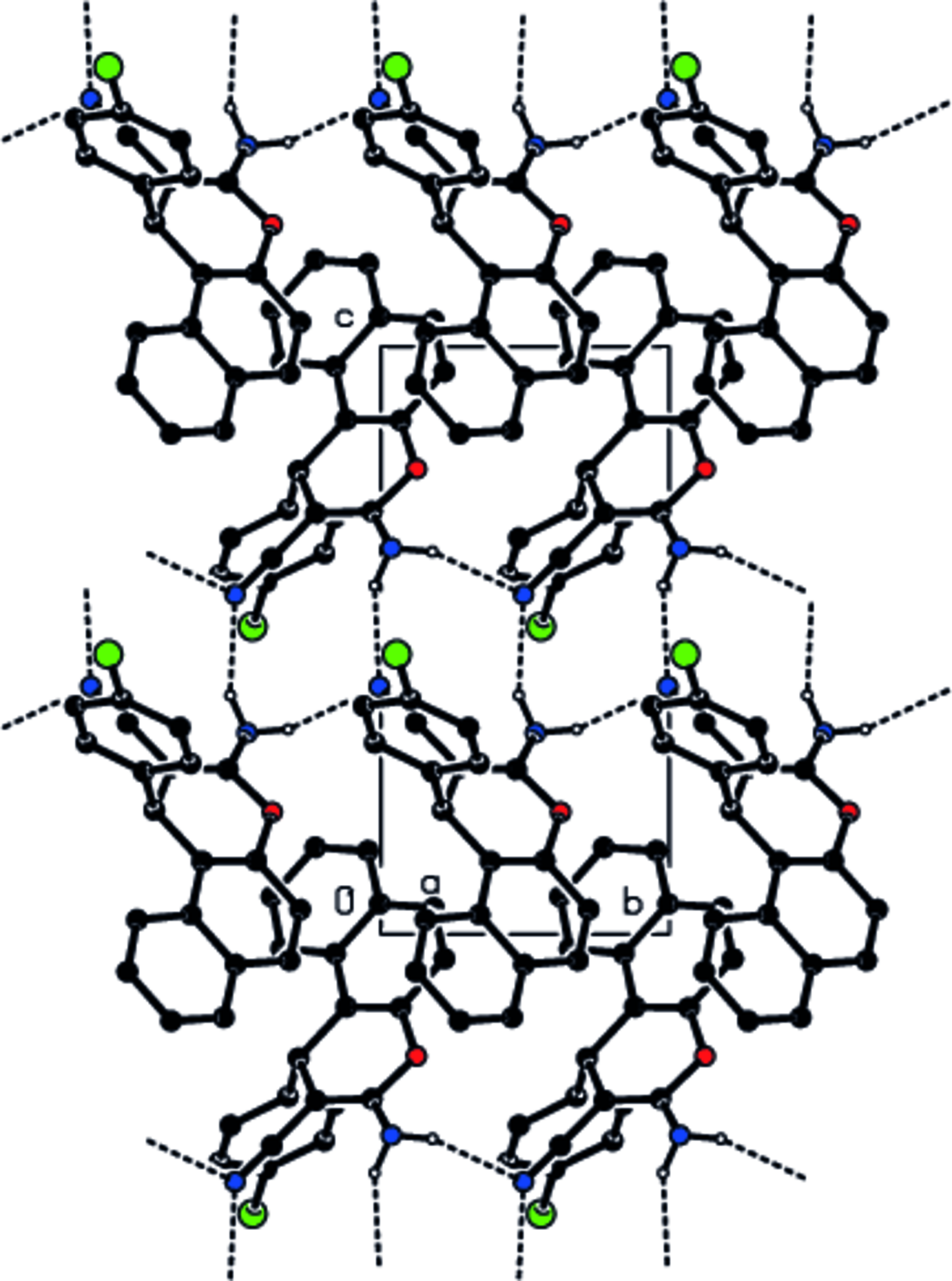

Supplement: Supplementary file 5 [file e-71-0o481-fig2.tif]

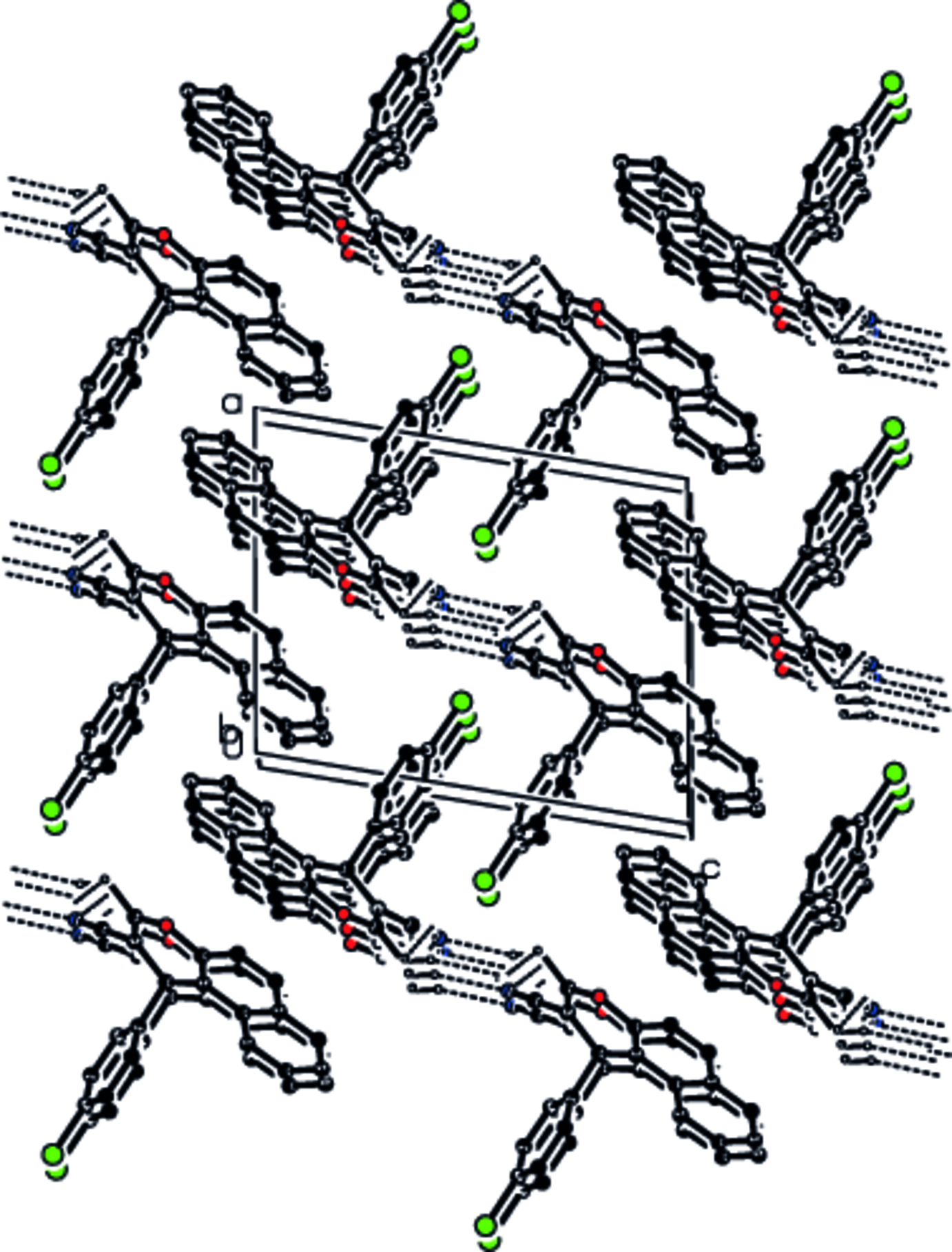

Supplement: Supplementary file 6 [file e-71-0o481-fig3.tif]
